# Evaluation of the Effectiveness of Post-Stroke Metformin Treatment Using Permanent Middle Cerebral Artery Occlusion in Rats

**DOI:** 10.3390/ph14040312

**Published:** 2021-04-01

**Authors:** Gintare Zemgulyte, Shigeru Tanaka, Izumi Hide, Norio Sakai, Katryna Pampuscenko, Vilmante Borutaite, Daiva Rastenyte

**Affiliations:** 1 Medical Academy, Department of Neurology, Lithuanian University of Health Sciences, A. Mickeviciaus Str. 9, LT-44307 Kaunas, Lithuania; daiva.rastenyte@lsmuni.lt; 2 Department Molecular and Pharmacological Neuroscience, Hiroshima University, 1 Chrome-2-3 Kasumi, Minami Ward, Hiroshima 734-8551, Japan; tanakamd@hiroshima-u.ac.jp (S.T.); ihide@hiroshima-u.ac.jp (I.H.); nsakai@hiroshima-u.ac.jp (N.S.); 3 Medical Academy, Neuroscience Institute, Lithuanian University of Health Sciences, Sukileliu 13, LT-50162 Kaunas, Lithuania; katryna.pampuscenko@lsmuni.lt (K.P.); vilmante.borutaite@lsmuni.lt (V.B.)

**Keywords:** metformin, post-stroke treatment, permanent middle cerebral artery occlusion, ischemia, microglia

## Abstract

Stroke is the second leading cause of death worldwide. Treatment options for ischemic stroke are limited, and the development of new therapeutic agents or combined therapies is imperative. Growing evidence suggests that metformin treatment, due to its anti-inflammatory action, exerts a neuroprotective effect against ischemia/reperfusion-induced brain damage. Experimental assessment has typically been performed in models of cerebral transient ischemia followed by long-term reperfusion. The aim of this study was to evaluate the neuroprotective effect of metformin treatment after permanent middle cerebral artery occlusion (pMCAO) without reperfusion in rats. Neurological deficits were assessed using the Longa scale, which offers a graded scale on body movement following pMCAO. Both infarct size and brain oedema area were measured by staining with 2,3,5-triphenyltetrazolium chloride. The number of neurons and total and activated microglia, as well as interleukin 10 (IL-10) production, in brain sections were evaluated by immunohistochemical staining. Our results show that metformin treatment improves the neurological state and reduces infarct size after 120 h of pMCAO. Metformin also prevents neuronal loss in the ischemic cortex but not in the striatum after 48 h of pMCAO. Moreover, post-stroke treatment with metformin significantly decreases the number of total and activated microglia at 48 h. The anti-inflammatory effect of metformin is associated with increased IL-10 production at 48 h after pMCAO. The results of the present study suggest that post-stroke treatment with metformin exerts anti-inflammatory and neuroprotective effects in a pMCAO model.

## 1. Introduction

Stroke is the second leading cause of death worldwide [1]. Reperfusion therapy, including intravenous thrombolysis with tissue plasminogen activator and mechanical thrombectomy, is the only approved treatment for acute ischemic stroke [2]. Since the time from stroke onset is a key determinant of patient selection for reperfusion therapy [3], only a portion of ischemic stroke patients are eligible for it [4]. Over the last three decades, various pharmacologic agents and non-pharmacological therapies have been evaluated to protect the brain from ischemic injury [5]. However, the results of studies with citicoline [6], excitatory amino acid modulators [7], magnesium [8] and different calcium channel blockers [9] concluded that there was no evidence of benefit. Therefore, the development of new neuroprotective agents or combined therapies is imperative [10].

Over the last four decades, a variety of animal stroke models have been developed with the aim of identifying the mechanisms that underlie cerebral ischemia and developing new therapies for ischemic stroke [11]. Currently, intravascular filament occlusion of the middle cerebral artery (MCA) in rodents is the most widely used model of focal brain ischemia, which was originally developed in rats [12]. The transient middle cerebral artery occlusion (MCAO) model mimics ischemic stroke with reperfusion [13], whereas permanent middle cerebral artery occlusion (pMCAO) mimics large vessel occlusion without a reperfusion process and is equally important. Permanent occlusion is more difficult to achieve and is associated with increased mortality attributable to both increased swelling and increased intracranial pressure [14]. On the other hand, medications tested in transient MCAO models rapidly reach the ischemic core and penumbra, producing their protective effects directly and immediately after reaching the ischemic zone. However, transitioning the drug into the pMCAO model leads to negative results [13]. In addition, in many patients, it is impossible to perform intravenous thrombolysis with tissue plasminogen activator as well as mechanical thrombectomy, and thus, long-term occlusion commonly develops as a consequence. There is a demand for more adequate models of permanent occlusion and for further investigations on how to apply treatment in these cases.

After ischemic brain injury, microglial cells undergo activation [15] and rapidly migrate toward the lesion site for clearing dead cells and restoring neuronal function [16]. On the other hand, excessive microglial activation [17] can cause irreversible neuronal damage resulting in exacerbation of tissue injury [15]. Numerous studies highlight the significance of neuroinflammatory processes in stroke pathogenesis, but experimental assessment has typically been performed in models of cerebral ischemia followed by reperfusion [18,19,20]. However, the involvement of such processes in brain injury occurring during severe permanent cerebral ischemia remains largely unknown [21] and needs further investigation in order to elucidate the role of microglia activation.

Metformin is a biguanide derivative that is widely used to treat type 2 diabetes [22]. Growing evidence suggests that metformin treatment also exerts neuroprotective effects against ischemia-induced brain damage [23]. The molecular mechanisms of action of metformin in the brain are not entirely clear but may be related to inhibition of neuronal death and suppression of inflammatory responses. Metformin administration has been shown to reduce markers of brain inflammation [24] in a cerebral ischemia/reperfusion model [25,26]. There is a lack of studies assessing the effects of poststroke treatment with metformin in the pMCAO model, particularly in protracted ischemia without reperfusion. Moreover, the mechanisms underlying the neuroprotective effects of metformin on neuronal survival are not entirely clear [24]. Thus, the aim of this study was to evaluate the neuroprotective effect of metformin treatment after pMCAO.

## 2. Results

### 2.1. The Effect of Metformin on Infarct Size, Brain Oedema and Neurological State after pMCAO

**Figure 1:**
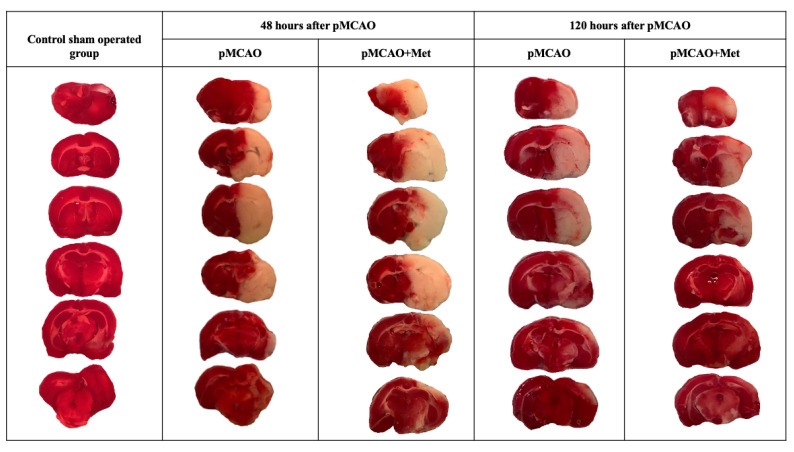
Representative TTC-stained brain sections from each group. The infarct area is white, and the normal area is red.

**Figure 2:**
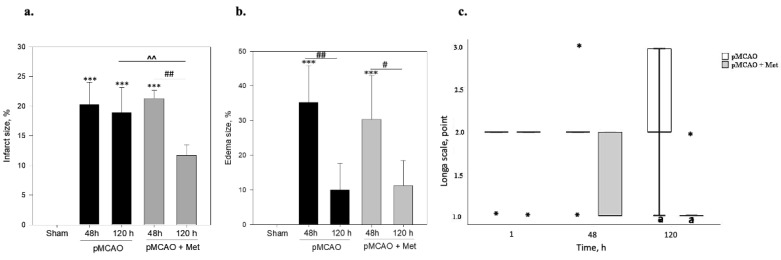
The effect of metformin on brain infarct size, brain oedema size and neurological state after pMCAO in rats. Metformin (Met 50 mg/kg/day) or saline were injected after pMCAO and repeated every day until sacrifice, which was 2 or 5 times based on time of stroke. (**a**) The infarct area was measured by TTC staining as described in the Methods section. One-way ANOVA followed by a Fisher LSD test was used to compare the effects. All bars represent mean ± SEM (4–5 independent experiments on individual animal), *** *p* < 0.001 compared to sham group, ## *p* < 0.01 pMCAO 48 h + Met compared to pMCAO 120 h + Met group, ^^ *p* < 0.01 pMCAO 120 h compared to pMCAO 120 h + Met group. (**b**) Brain oedema size was measured by TTC staining as described in the Methods section. One-way ANOVA followed by a Fisher LSD test was used to compare the effects. All bars represent mean ± SEM (4–5 independent experiments on individual animal), *** *p* < 0.001 compared to sham group, # *p* < 0.05 pMCAO 48 h + Met compared to pMCAO 120 h + Met group, ## *p* < 0.05 pMCAO 48 h compared to pMCAO 120 h group. (**c**) Neurological state was evaluated by Longa test as described in the Methods and Materials section. The neurological state of sham-operated animals according to the Longa scale was 0 points. The Mann–Whitney test was used to compare the effects. The boxplot of neurological state represents median [IQR] (5 independent experiments on individual animals), *-outliers, the minimum value, the first quartile, the median, the third quartile, the maximum value, ^a^ *p* = 0.05 pMCAO 120 h compared to pMCAO 120 h + Met group.

Infarct size and area of brain oedema at 48 h and 120 h after pMCAO were measured by TTC staining. TTC staining was performed in the control sham operated group after sham operation. As demonstrated in Figure 1, the sham group showed no infarction, while extensive infarction was observed for different groups after pMCAO. As noted in Figure 2a, in the pMCAO group without metformin, infarct size was similar at 48 and 120 h after occlusion. In rats receiving metformin, infarct size was similar to that noted in the pMCAO without metformin group 48 h after occlusion (Figure 2a). However, at 120 h, infarct size in metformin-treated rats decreased by 55% (*p* = 0.008) compared to the pMCAO group with metformin at 48 h and was reduced by 38% (*p* = 0.016) compared with the pMCAO group without metformin after 120 h pMCAO (Figure 2a). The area of brain oedema in the pMCAO group without metformin and metformin-treated groups was similar at 48 h pMCAO and substantially decreased after 120 h in both groups. However, no statistically significant differences were noted between the pMCAO group and metformin-treated groups either after 48 h or after 120 h of pMCAO (Figure 2b). The effect of metformin on neurological deficits was examined using the Longa scale and measured at 1, 48 and 120 h after permanent MCAO. A significant difference in neurological deficits was noted between the pMCAO group with metformin and the pMCAO group without metformin at 120 h after pMCAO (Figure 2c). 

### 2.2. The Effect of Metformin on Neuronal Densities in Brain Sections after pMCAO

**Figure 3:**
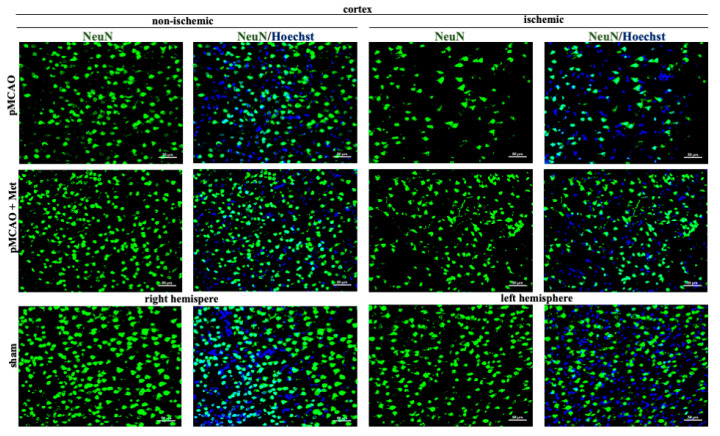
Representative images of immunohistochemically stained neurons in the cerebral cortex. Brain slices were prepared at 48 h after pMCAO with or without metformin (50 mg/kg) and then stained with anti-NeuN antibody as described in Methods. Images were obtained using fluorescence microscopy (Olympus IX71S1F-3, Orangeburg, NY, USA) at 20× magnification. Scale bars 50 µm.

**Figure 4:**
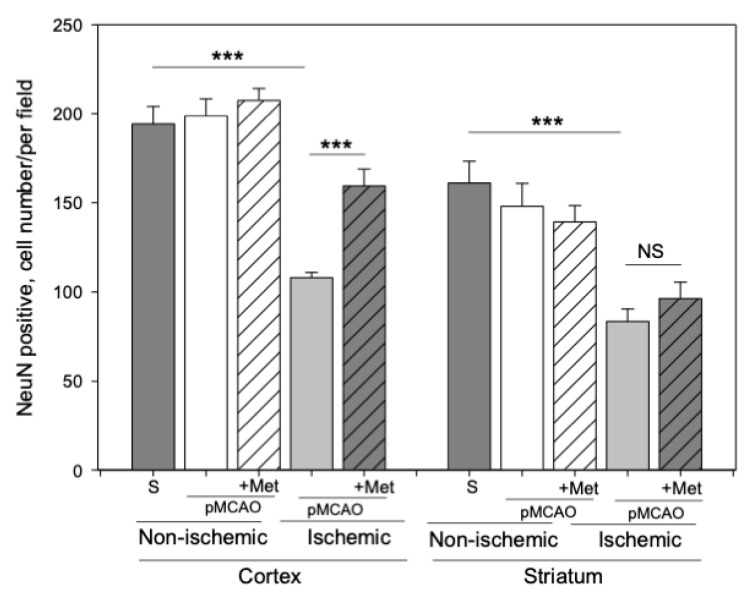
The effect of metformin treatment on the number of neurons (NeuN) after pMCAO. Brain slices were prepared at 48 h after pMCAO with or without metformin (50 mg/kg) and then stained with anti-NeuN antibody as described in Methods. Images were analyzed by fluorescence microscopy (Olympus IX71S1F-3, Orangeburg, NY, USA) at 20× magnification. Data expressed as mean number of cells per field. One-way ANOVA followed by a Fisher LSD test was used to compare the effects. All bars represent mean ± SEM (3–4 independent experiments on individual animals). *** *p* < 0.001, NS—not significant.

Neurons within the ischemic core rapidly degenerate, but in the surrounding ischemic penumbra neuronal death occurs with a delay [27]. Thus, we evaluated whether metformin treatment protects against pMCAO-induced neuronal loss in the striatum (core) and cortex (penumbra). Representative images of cortical neurons stained with NeuN antibodies (a specific marker for mature neurons) in brain sections 48 h after pMCAO are provided in Figure 3. As demonstrated in Figure 4, the number of neurons in the sham group was 171 ± 10 cells/field in the striatum and the number of neurons in the cortex was 205 ± 10 cells/field. The number of neurons in the non-ischemic hemisphere of pMCAO remained the same as in sham-operated animal brains, whereas in the ischemic hemisphere the number of neurons decreased to 83 ± 7 cells/field in the striatum and to 108 ± 3 cells/field in the cortex. In the ischemic hemisphere, treatment with metformin resulted in an increased number of neurons in the cortex (159 ± 9 cells/field), *p* < 0.001, while in the striatum, it remained unchanged at 96 ± 9 cells/field (Figure 4). Metformin had no effects on cortical and striatal neuronal numbers in the non-ischemic hemisphere (Figure 4). The data suggest that post-occlusion treatment with metformin prevented neuronal loss in the penumbra but not in the ischemic core.

### 2.3. The Effect of Metformin on Microglial Activation and IL-10 Production after pMCAO

**Figure 5:**
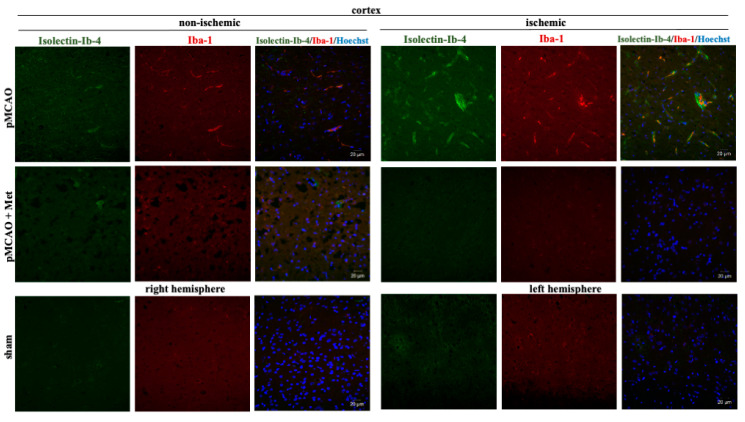
Representative images of immunohistochemically stained microglia in the cerebral cortex. Brain slices were prepared at 48 h after pMCAO with or without metformin (50 mg/kg) as described in Methods. Microglia were co-labeled with anti-Iba1 antibody (activated; red) and Isolectin-Ib4 (total; green), cell nuclei were stained with Hoechst33342. Images were obtained using confocal laser-scanning microscope LSM 700 with ZEN 2010 software (Carl Zeiss, Jena, Germany) at 40× magnification. Scale bars 20 µm.

**Figure 6:**
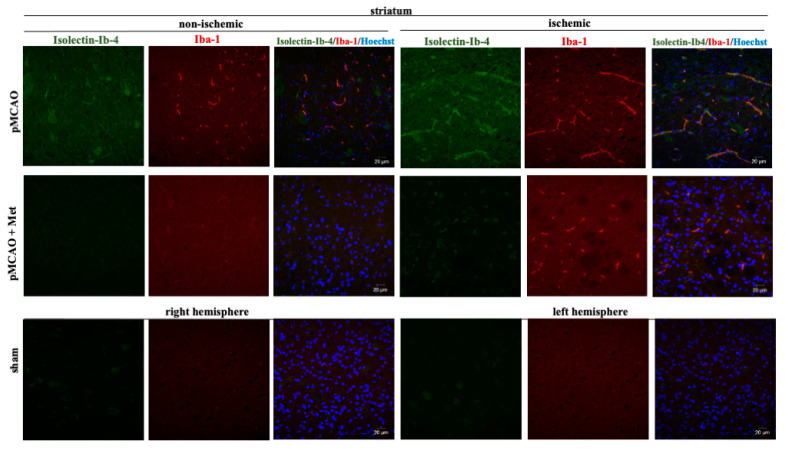
Representative images of immunohistochemically stained microglia in the cerebral striatum. Brain slices were prepared at 48 h after pMCAO with or without metformin (50 mg/kg) as described in Methods. Microglia were co-labeled with anti-Iba1 antibody (activated; red) and Isolectin-Ib4 (total; green), cell nuclei were stained with Hoechst33342. Images were obtained using confocal laser-scanning microscope LSM 700 with ZEN 2010 software (Carl Zeiss, Jena, Germany) at 40× magnification. During analysis of images using ImageJ software, the background was subtracted. Scale bars 20 µm.

**Figure 7:**
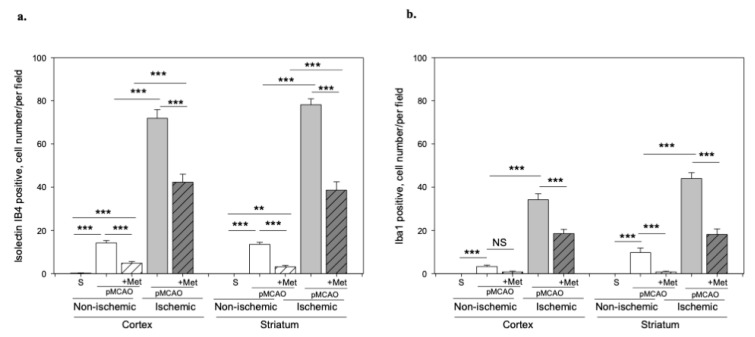
The effect of metformin treatment on number of microglia after pMCAO. (**a**) Number of total microglia (Isolectin-Ib4-positive), (**b**) Number of activated microglia (Iba-1-positive). Brain slices were prepared at 48 h after pMCAO with or without metformin (50 mg/kg) and then fluorescently labeled isolectin-IB4 and stained with anti-Iba1 antibody as described in Methods. Data are expressed as mean number of cells per field. One-way ANOVA followed by an LSD test was used to compare the effects. All bars represent mean ± SEM (3–4 independent experiments on individual animal). *** *p* < 0.001, ** *p* < 0.01, NS—not significant.

It is known that the pathogenesis of brain ischemic injury is closely linked to inflammatory processes. Thus, we evaluated the effect of metformin on microglia proliferation and activation (which are hallmarks of neuroinflammation) at 48 h of pMCAO. Total and activated microglia were labelled with isolectin-GS-Ib4 (a marker for brain microglia) and anti-Iba-1 (antibodies against ionized calcium–binding adaptor molecule 1, a specific marker for activated microglia), respectively. Representative images of total and activated microglia at 48 h of pMCAO are shown in Figure 5 and Figure 6. In the sham group, low numbers of microglia (about 6 cells/field) were seen in the striatum and cortex; however, in the ischemic hemisphere, the total number of microglia increased up to 71 ± 4 cells/field in the cerebral cortex and up to 78 ± 3 cells/field in the striatum. The number of activated microglia was 34 ± 3 and 44 ± 3 cells/field in the cortex and striatum, respectively (Figure 7a,b). After treatment with metformin, the number of isolectin-IB-4-positive cells decreased to 42 ± 4 and 39 ± 4 cells/field, and the number of activated microglia decreased to 19 ± 2 and 18 ± 3 cells/field in the cortex and striatum of the ischemic hemisphere, respectively (Figure 7a,b). Notably, in the non-ischemic hemisphere, the total numbers of microglia were increased in both cortex and striatum, though to a lesser extent than in the ischemic hemisphere (up to about 14–16 cells/field), and after treatment with metformin, a substantial decrease in microglial numbers was observed (Figure 7a). In contrast, the number of activated Iba1-positive microglia in the non-ischemic hemisphere after pMCAO was increased only in the striatum (10 ± 2 cells/field) and was decreased to a negligible level (about 1 cell/field) after treatment with metformin (Figure 7b). Altogether, the results suggest that metformin treatment inhibits pMCAO-induced microglial proliferation and activation.

**Figure 8:**
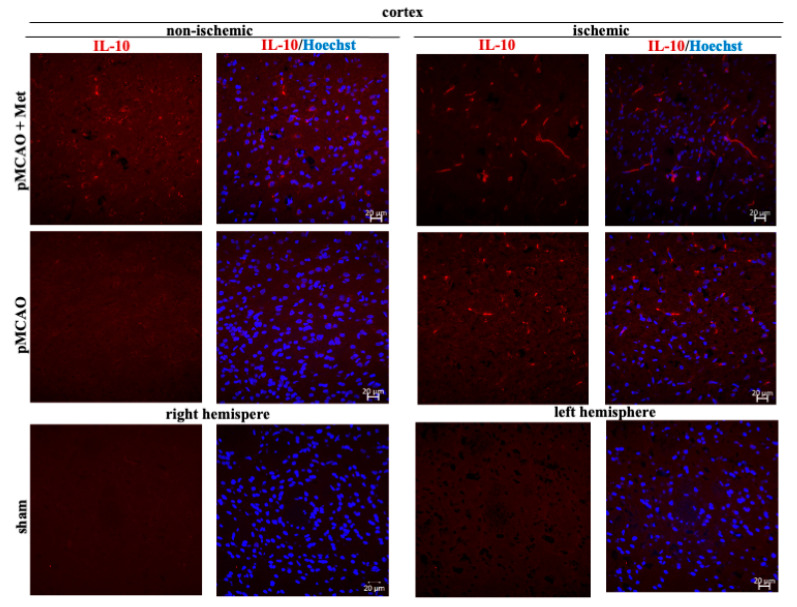
Representative images of immunohistochemically stained brain slices against IL-10 in cerebral cortex. Brain slices were prepared at 48 h after pMCAO with or without metformin (50 mg/kg) and stained with anti-IL10 antibody as described in Methods. Images were obtained using confocal laser-scanning microscope LSM 700 with ZEN 2010 software (Carl Zeiss, Jena, Germany) at 40× magnification. Scale bars 20 µm.

**Figure 9:**
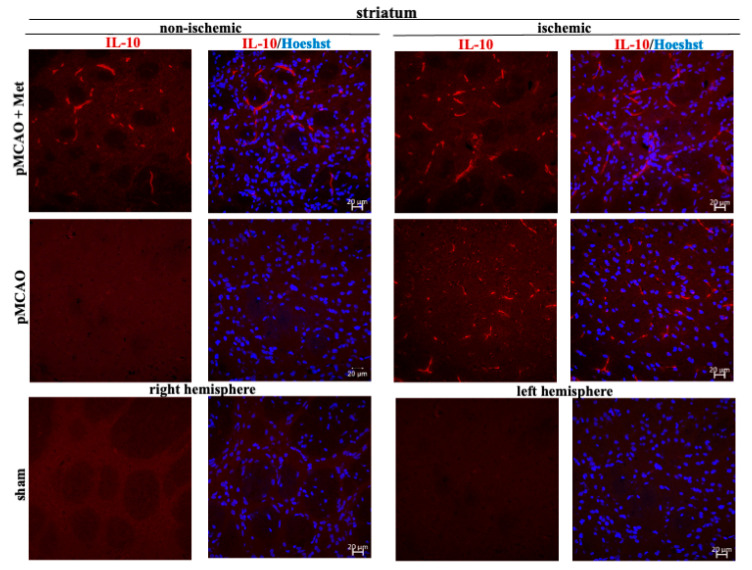
Representative images of immunohistochemically stained brain slices against IL-10 in cerebral striatum. Brain slices were prepared at 48 h after pMCAO with or without metformin (50 mg/kg) and stained with anti-IL10 antibody as described in Methods. Images were obtained using confocal laser-scanning microscope LSM 700 with ZEN 2010 software (Carl Zeiss, Jena, Germany) at 40× magnification. Scale bars 20 µm.

**Figure 10:**
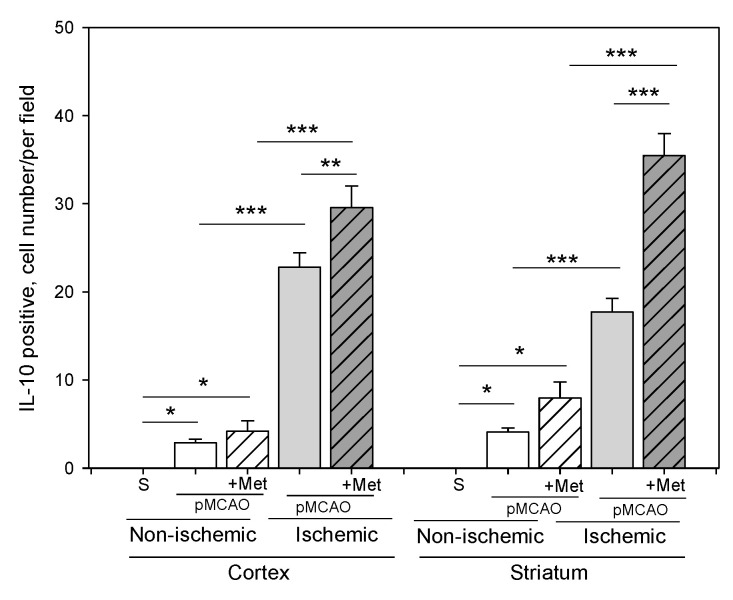
The effect of metformin treatment on IL-10 production after pMCAO. Brain slices were prepared at 48 h after pMCAO with or without metformin (50 mg/kg) and then stained with anti-IL-10 antibody as described in Methods. Data expressed as mean number per field. One-way ANOVA followed by a Fisher LSD test was used to compare the effects. All bars represent mean ± SEM (3–4 independent experiments on individual animals). *** *p* < 0.001, ** *p* < 0.01, * *p* < 0.05.

Microglia/macrophages react quickly to defend the brain against injury by acquiring distinct functional phenotypes: the M1 and M2 polarization phenotypes. Different microglia/macrophage phenotypes may have distinct roles because the M1 population is mainly destructive, while the M2 population is neuroprotective [16]. Since IL-10 is an additional marker of the M2 phenotype of microglia, we investigated whether metformin treatment has an effect on production of the anti-inflammatory cytokine IL-10. The results of immunohistochemical staining of brain slices using an anti-IL-10 antibody are shown in ischemic and non-ischemic hemisphere of the cortex and striatum (Figure 8 and Figure 9). As demonstrated in Figure 10, there was no production of IL-10 in the striatum and cortex in the sham group. In contrast, in the ischemic hemisphere of brains after pMCAO, numbers of cells with IL-10 were substantially increased, reaching 23 ± 2 and 18 ± 2 cells/field in the cortex and striatum, respectively. Treatment with metformin further increased IL-10 staining up to 30 ± 2 and 35 ± 2 cells/field in the cerebral cortex and striatum of the ischemic hemisphere, respectively (Figure 10). The amount of IL-10 in the non-ischemic hemisphere after occlusion also increased but to a lesser extent in both cortex and striatum and reached 3–4 numbers/field; however, treatment with metformin had no effect on the amount of IL-10 (Figure 10). These results suggest that metformin may suppress ischemia-induced neuroinflammation via stimulation of IL-10 production in microglia. 

## 3. Discussion

Worldwide, several studies have been performed in which metformin preconditioning before pMCAO has been used [28,29,30], while research using pMCAO and post-stroke treatment with metformin is lacking. The present study aimed to assess the neuroprotective effect of post-occlusion treatment with metformin using the model of protracted pMCAO in rats, which resembles to some extent clinical situations when thrombolysis or thrombectomy is not possible. The main novel and important finding of this study was that metformin applied after the onset of stroke exhibits neuroprotective effects in the acute phase by reducing neuroinflammation and neuronal loss and in the sub-acute phase of ischemic stroke by reducing the infarct size and neurological deficit 120 h post-occlusion.

A major contributing factor of experimental research assessing post-stroke treatment is the timeframe of the metformin treatment following cerebral occlusion [24]. Considering infarct size, brain oedema and neurological deficits, this study did not identify statistically significant differences between the MCAO group and the MCAO with metformin group at 48 h after pMCAO. However, post-occlusion treatment with metformin significantly decreased the infarct size and ameliorated the neurological state at 120 h of ischemic stroke. The decrease of the infarct size may be associated with increased vascular remodeling in the stroke area and shrinkage of the infarct. In studies by other investigators, the neuroprotective effect of metformin has been shown to be dependent on the onset time of treatment and the duration of metformin administration [31,32]. It has been demonstrated that chronic metformin preconditioning significantly reduced infarct volume and attenuated neurological deficits at 24 h and 96 h after pMCAO in rats with metformin doses similar to those used in the present study [28]. The advantage of our study was that the neuroprotective effect of metformin was observed after post-stroke application; thus, such treatment exhibits more significant relevance to clinical practice than metformin preconditioning.

The molecular mechanisms of action of metformin in the brain are not entirely clear but may be related to inhibition of ischemia-induced neuronal death and suppression of inflammatory responses. Ischemia causes mitochondrial damage and dysfunction, opening the mitochondrial permeability transition pore, leading to cell death [33]. Thus, it has been suggested that metformin may exert neuroprotective effects by inhibiting mitochondrial respiratory chain complex I, which has been shown to increase mitochondrial calcium retention capacity and prevent opening of the mitochondrial permeability transition pore [34,35]. However, there is a scarcity of studies in vivo evaluating modulation of mitochondrial function by metformin in the stroke area. Emerging evidence also indicates that the beneficial effect of metformin may be associated with activation of AMPK [36], which may lead to enhanced neuronal survival [29]. However, these mechanisms involving activation of the AMPK cascade are more likely to be promoted during pre-conditioning with metformin rather than during post-ischemic application, as ischemia by itself may activate AMPK. Further investigation into the mechanism of action of metformin at cellular and molecular levels is needed to fully elucidate the precise neuroprotective mechanism of metformin treatment.

The timeframe of the metformin treatment and the dose of metformin may be the cause of the different research results [24]. In various studies, metformin doses tended to range from 10 mg/kg/day to 500 mg/kg/day with different administration routes (gavage and intraperitoneal) in MCAO models [29,37]. It has been shown that pre-treatment with a single 10 mg/kg/day dose of metformin significantly reduced infarct volume and neurological deficits after 24 h of pMCAO [29]. In another study, it has been shown that 10 mg/kg/day metformin significantly counteracted ischemic injury in a time-dependent manner: the most effective neuroprotection was noted within 7 days of pre-treatment, whereas other doses were not effective [30].

The present study has identified that post-occlusion treatment with metformin decreased proliferation and activation of microglia and increased production of the anti-inflammatory cytokine IL-10 in the cortex and striatum of the ischemic hemisphere. The less significant delayed brain damage caused by activated microglia may be due to a decrease of active microglial cells in the stroke area. Note that metformin suppressed stimulation of microglial proliferation in the cortex and striatum, while decreased activation of microglia was only observed in the striatum of the non-ischemic hemisphere. Thus, the attenuation of activated microglia by metformin and developed smaller delayed lesions can be defined as a beneficial effect. In line with our findings, it has been found that post-stroke chronic metformin injection switched the ischemia/reperfusion-induced pro-inflammatory phenotype of microglia/macrophages towards an anti-inflammatory phenotype via the AMPK-dependent pathway [25]. In a permanent MCAO model using chronic preconditioning with metformin, it has been demonstrated that metformin suppressed NF-kB activity and cytokine (TNF-α, IL-1β, IL-6) production [28]. There is evidence that metformin inhibits inflammasome activation [38,39] and decreases phosphorylation of c-JNK-1 [40,41]. In addition, it has been shown that metformin-induced AMPK-dependent or independent mTOR inhibition switches the M1 phenotype of macrophages towards the M2 phenotype [42]. It is not entirely clear how metformin stimulates IL-10 production in ischemia-affected brains. It has been suggested that metformin can promote IL-10 production in LPS-treated macrophages by acting on complex I of the mitochondrial respiratory chain [43]. Whether a similar mechanism may operate in metformin-treated microglia during ischemia needs to be further investigated. Our results, in line with previously published studies [25], suggest that metformin-inhibited microglial activation and enhanced IL-10 production may contribute to the beneficial effects of metformin therapy on stroke outcome. Interestingly, metformin treatment has also been shown to be effective against other, non-neurological disorders (such as non-alcoholic fatty liver disease, dysfunction of pancreatic ß cells and cancer), the pathogenesis of which involves inflammatory processes [44].

The important finding of the present study was that treatment with metformin prevented ischemia-induced neuronal loss in the cortex but not in the striatum of the ischemic hemisphere. After ischemic stroke, a large number of neurons die acutely due to reduced blood flow causing energy depletion [27]. Fifield et al. [45] found that the largest loss of neurons occurred during the first 4 h post-stroke in the non-perfused core and in the hypo-perfused region, but numbers of neurons were similar between 8 h and 72 h after stroke in the perfused core or the hypo-perfused region. However, in the penumbra surrounding the infarct core, delayed neuronal cell death may occur [45]. It has been shown that delayed death of neurons may be related to activation of microglia leading to primary phagocytosis of neurons [46,47]. Thus, the neuroprotective effect of metformin in the cortex may be due to decreased activation of microglia resulting in suppression of neuronal loss. Metformin did not prevent loss of neurons in the striatum of stroke, because in this area neurons may die before activation of microglia.

It is important to mention that recent clinical studies revealed that pre-treatment with metformin resulted in less severe strokes, beneficial thrombolysis outcome and reduced neurological deficits in diabetic patients [48,49]. Thus, these results suggest that metformin has a neuroprotective effect, although there is a lack of post-ischemic treatment studies in non-diabetic patients. On the other hand, according to Sardu and colleges [50], metformin may reduce the risk of cardiovascular disorders in pre-diabetic patients. This strengthens the possibility that clinical application of metformin could be reliable in the general population as well.

The present study was the first to evaluate the impact of metformin after continued treatment for 48 and 120 h after MCAO in rats. However, the study has some limitations that should be taken into consideration. In the present study, neurological state was evaluated using the Longa scale, a test which assesses only motor deficits; however, the functioning of other neural systems was not examined. Furthermore, metformin was administered 1 h after pMCAO; thus, it would be complicated to implement in a real clinical situation. Consequently, future studies are necessary to assess the effect of metformin in delayed treatments that are clinically relevant to stroke therapies. A single dose of metformin was injected intraperitoneally to avoid metabolism in the gastrointestinal tract; however, in clinical situations, metformin is administered orally. Therefore, different doses of metformin and various routes of administration should be evaluated in further experiments as well.

## 4. Methods and Materials

### 4.1. Middle Cerebral Artery Occlusion

Experiments were performed and approved by the Animal Care and Use Committee, Hiroshima University (A18-105) and by the State Food and Veterinary Service of Lithuania (No. G2-79). Adult male Wistar rats (200–280 g, 7–8 weeks) were used in this study. Animals were maintained in ventilated cages in a standard animal room under a 12 h light/dark cycle and allowed free access to food and water. During the surgical procedure, rats were anaesthetized with sevoflurane or isoflurane in air/oxygen (70:30%) using a facemask. Rectal temperature was maintained at approximately 36.5 ± 0.5 °C with a circulating heating pad and a heat lamp. Subsequently, the left common carotid artery (CCA), external carotid artery (ECA) and internal carotid artery (ICA) were exposed through a midline incision. Then, both the CCA and the ICA were isolated and carefully separated from the adjacent vagus nerve. The CCA and the ECA were tied using 5-0 silk sutures. In addition, a microvascular clip was placed across the bifurcation of the CCA. A 4-0 nylon monofilament with a heat-blunted tip was gently inserted into the left ICA via the CCA until the tip of the filament reached the origin of the MCA, and special care was taken not to enter the pterygopalatine artery. After surgery all animals were held in individual cages. The filament was left until the rat was sacrificed.

### 4.2. Animal Groups

**Figure 11:**
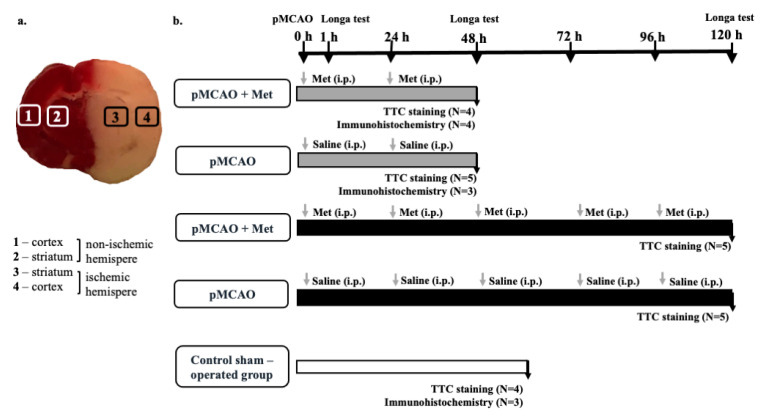
(**a**) Partition of brain slice by TTC staining at 48 h after pMCAO using immunohistochemical staining. (**b**) Schematic illustration of the experiment.

Rats were divided into the following groups: rats receiving intraperitoneal metformin (Sigma-Aldrich, St. Louis, MO, USA, LOT: LRAB3694) injections (50 mg/kg/day) (pMCAO group with metformin) or equal volumes of saline injections (pMCAO group). Rats were sacrificed after 48 h or 120 h post-occlusion. The control sham-operated group was subjected to skin incision only. Injection of metformin or saline was initiated immediately after pMCAO and was repeated every day until sacrifice, which was 2 or 5 times depending on stroke onset. Figure 11b presents a schematic illustration of experiments and the number of animals in each group. 

### 4.3. Measurement of Infarct Size

The animals were euthanized via CO_2_ inhalation at 48 or 120 h after occlusion. Their brains were subsequently removed. Brains were cut with a brain matrix at a thickness of 2 mm into 6 sections. The brain sections were incubated in 2% 2,3,5-triphenyltetrazolium chloride (TTC, Sigma) at 37 °C for 30 min. The infarct region was defined as the area with reduced staining within the MCA area. The size of the brain injury was measured using ImageJ/Fiji 1.46 software (the National Institutes of Health and the Laboratory for Optical and Computational Instrumentation, London, UK). The percentage of a total infarcted size was calculated by dividing the infarct size by the total size of the bilateral hemisphere of six consecutive coronal sections. To correct for tissue swelling, the infarct size was calculated using the equation described by Boyko et al. [51]: corrected infarct size = infarct size × contralateral hemisphere size/ipsilateral hemisphere size.

### 4.4. Measurement of Brain Oedema Area

The brain oedema area was assessed and calculated in arbitrary units (pixels) from the summation of coronal slice areas using ImageJ/Fiji 1.46 software. The brain oedema area was expressed as a percentage of the normal areas in the contralateral unaffected hemisphere [52]. 

### 4.5. Behavioural Testing

The Longa test was used to assess the neurological status at 1, 48 and 120 h after the onset of occlusion. The neurologic findings were scored on a five-point scale: 0 points—no neurological findings; 1 point—failure to extend right forepaw completely; 2 points—circling to the right; 3 points—falling to the right; and 4 points—no spontaneous walking and exhibiting depression [53].

### 4.6. Immunohistochemistry

The rats were anesthetized with Xylasine 2% (Bela-pharm, Vechta, Germany, H1125-02) 10 mg/kg and Ketamine (Vetoquinol, Lure, France, LOT: 5A1792M) 75 mg/kg by intraperitoneal injection. Subsequently, animals were transcardially perfused with phosphate-buffered saline (PBS, Gibco, Paisley, UK) followed by 4% paraformaldehyde (Merck, Darmstadt, Germany) in PBS. The brains were collected, fixed in 4% paraformaldehyde and were treated with 30% sucrose solution. According to Herman and Watson [54], the brain blocks surrounding the ischemic lesion (+1.2 to −0.26 mm from bregma) were cut into 16-µm-thick coronal sections with a cryostat (Cryostat NX50, Thermo Scientific, Waltham, MA, USA). The sections were blocked with 5% bovine serum albumin or 5% goat serum in PBS supplemented with 0.5% TritonX-100. The sections were incubated with the following primary antibodies: goat anti-Iba1 (Invitrogen by Thermo Fisher Scientific, Waltham, MA, USA, 1:100), rabbit anti-IL10 (Invitrogen by Thermo Fisher Scientific, 1:500) and mouse anti-NeuN (Invitrogen by Thermo Fisher Scientific, 1:500) overnight at 4 °C. Then, brain sections were incubated with anti-goat/anti-rabbit or anti-mouse secondary antibodies conjugated with Alexa Fluor594 or AlexaFluor488 (Invitrogen by Thermo Fisher Scientific, 1:200) for 2 h at room temperature, respectively. Microglial cells were labelled with 1 µg/mL Isolectin GS-IB4 from *Griffonia simplicifolia* and Alexa Fluor488 conjugate (Invitrogen by Thermo Fisher Scientific). Cell nuclei were stained with Hoechst 33342 (10 µg/mL). Brain sections were mounted with ProLong Gold antifade reagent (Invitrogen by Thermo Fisher Scientific).

### 4.7. Fluorescence and Cell Quantification

At least two histological brain sections per animal were used for each staining to detect isolectin-GS-IB4-, Iba-1-, IL-10- and NeuN-positive cells. Stained brain sections were analyzed by fluorescence microscopy (Olympus IX71S1F-3, Orangeburg, NY, USA) at 20× magnification. Four brain regions (ischemic and non-ischemic cortex and striatum) were analyzed in each experiment on an individual animal (Figure 11a), and cells were counted in five randomly selected microscopic fields in each brain region on the histological section. Calculation of average cell numbers were performed using ImageJ program. Data were expressed as mean number of cells per field. Analysis of sections was performed by an independent researcher to avoid bias in assessing the protective effect of treatment. Representative images were obtained using the confocal laser-scanning microscope LSM 700 with ZEN 2010 software (Carl Zeiss, Jena, Germany) at 40× magnification.

### 4.8. Statistical Analysis

Statistical analyses were conducted using Sigma Plot 11.0 version software (Systat Software, San Jose, CA, USA) and SPSS 22.0 (Statistical Package for the Social Sciences for Windows, Chicago, IL, USA). Variables with normal distribution were presented as mean ± standard error (SEM); non-normally distributed variables were reported as median (interquartile range [IQR]). Statistical comparisons between independent groups were performed using one-way ANOVA (normality tested by Shapiro–Wilk test) followed by Fisher LSD test. The Mann–Whitney test was used to compare neurological scores in independent groups of animals. *p* ≤ 0.05 was considered statistically significant.

## 5. Conclusions

Post-occlusion treatment with metformin reduces microglial activation and neuronal loss in the penumbra but has no effect on the infarct size and neurological deficits at 48 h after pMCAO. At 120 h after pMCAO, treatment with metformin decreases the infarct size and ameliorates neurological state, suggesting that post-stroke treatment with metformin has neuroprotective effects.

## Data Availability

The data that support the findings of this study are study are available from the corresponding author upon request.

## Abbreviations

AMPK	AMP-activated protein kinase
CCA	common carotid artery
ECA	external carotid artery
IBA-1	ionized calcium-binding adaptor molecule 1
ICA	internal carotid artery
IL-10	interleukin—10
MCAO	middle cerebral artery occlusion
Met	metformin,
NeuN	neuronal nuclear antigen
PBS	phosphate-buffered saline
pMCAO	permanent middle cerebral artery occlusion
TTC	2,3,5-triphenyltetrazolium chloride
